# Anthropogenic environment and the burden of infectious gastrointestinal diseases in preschool children in Pacific Island countries, territories, and States: a narrative review

**DOI:** 10.1080/16549716.2026.2750012

**Published:** 2026-10-22

**Authors:** Jin Cao, Fan Yu, Harriet Lawford, Simon Toze, Benn Sartorius, Paul Jagals

**Affiliations:** a Frazer Institute, Faculty of Health, Medicine, and Behavioural Sciences, The University of Queensland, Brisbane, Australia; b Australian Centre for Water and Environmental Biotechnology, The University of Queensland, Brisbane, Australia; c School of Public Health and Primary Care, Fiji National University, Suva, Fiji; d Child Health Research Centre, The University of Queensland, Brisbane, Australia

**Keywords:** Infectious gastrointestinal diseases, children five and under, Pacific Islands region, disease burden, environmental risk factor

## Abstract

Infectious gastrointestinal diseases (I-GID) remain a major global burden among preschool children, influenced by the anthropogenic environment (AE) where children live and play. In Pacific Island Countries, Territories, and States (PICTS), remoteness, extreme climates, and resource constraints exacerbate AE conditions that facilitate I-GID transmission, but the actual burden and key AE risk factors remain unclear. This narrative review synthesised existing evidence on the I-GID burden and AE risk factors among preschool children in PICTS by searching four scientific databases, supplemented by grey literature. Reports were screened and graded by relevance to the review objectives (Highly relevant, Relevant, Low relevance, or Not relevant), with Not relevant reports excluded. We identified 48 reports from 12 PICTS published in 1972–2024; only six (12.5%) were highly relevant, and 15 (31.3%) were from Papua New Guinea. Thirty‑three (68.8%) described I-GID burden, but heterogeneous case definitions and metrics limited comparability, with estimates varying across settings and I-GID types. Twenty‑two (45.8%) examined AE risk factors, with preschool-specific evidence spanning physical environmental conditions and exposure-related behaviours, although findings for several commonly reported factors were inconsistent. Eight (16.7%) examined contextual factors, but none formally assessed whether these factors modified, mediated, or amplified AE–I-GID relationships. Overall, the evidence was limited and geographically uneven. I-GID burden among preschool children in PICTS, its AE risk factors, transmission pathways, and the role of contextual factors remain inadequately characterised. Integrated environmental, microbiological, epidemiological, and surveillance studies are needed to strengthen burden estimation, characterise transmission pathways, and identify priority AE and contextual risk factors for prevention and control.

## Paper Context

- **Main findings**: Across Pacific Island settings, evidence on the burden of I-GID and anthropogenic environmental factors among preschool children remains sparse and fragmented, with little on transmission pathways or how the factors interact with broader determinants.
- **Added knowledge**: This is the first narrative review to systematically map both the burden of I-GID and anthropogenic environmental risk factors specific to preschool children across Pacific Island settings, exposing critical gaps in their economic and health burden and in how these risk factors interact with and are influenced by broader factors in this environmentally vulnerable region.
- **Global health impact for policy and action**: This review highlights the urgent need for integrated studies that can deliver reliable burden estimates and identify priority risk factors to inform effective policy and action in resource-constrained Pacific Island settings.

## Background

Anthropogenic environments (AE) are human-built settings where people live, work, and interact. AE play an important role in the transmission of infectious gastrointestinal diseases (I-GID), which affect the gastrointestinal system and are primarily caused by microbial pathogens transmitted through environmental faecal-oral pathways [1,2]. Evidence suggests that I-GID are strongly associated with AE risk factors such as microbially contaminated water and food, inadequate sanitation, and poor environmental hygiene practices [3–7].

Preschool children (aged 5 years and under) are particularly vulnerable to AE risk factors due to their exposure behaviours (e.g. hand-to-mouth) and immunological immaturity [3,4,8–10]. Globally, I-GID ranks as the third leading cause of death among preschool children and accounts for 30.9 million disability-adjusted life years (DALYs) lost, with more than 60% caused by AE risk factors [7]. Notably, the burden of I-GID in preschool children is disproportionately higher in low- and middle-income countries (LMICs), ranging from 11 to more than 150 deaths per 100,000 across LMICs, with less than 1 death per 100,000 in high-income countries (HICs) [7]. This disparity is largely linked to lower economic development, which can exacerbate exposure to AE risk factors [11].

Within LMICs, Pacific Island Countries, Territories and States (PICTS) represent geographically dispersed and resource-limited environments where AE may be particularly vulnerable due to remoteness, small populations, variable economies, traditional customs and beliefs, and climate-related pressures [10,12–19]. These contextual characteristics may further increase preschool children’s exposure to AE risk factors and contribute to a greater burden of I-GID [8,14,20]. For example, floods, droughts, and tropical cyclones can damage housing and water and sanitation systems, reduce access to safe water, and increase environmental contamination, thereby facilitating faecal–oral transmission of I-GID. Broader socioeconomic conditions also directly shape AE risks through inequalities in household resources, infrastructure, and access to health services, or modify the extent to which climate-related pressures translate into AE risks.

Although efforts exist to protect preschool children from I-GID in PICTS, including Sustainable Development Goal (SDG) targets related to improving water, sanitation, and hygiene (WASH) and reducing under-five mortality [21], and longstanding support from the World Health Organization (WHO) and the United Nations Children’s Fund (UNICEF) [22,23], evidence on the burden of I-GID and its AE risk factors in PICTS remains limited. At present, evidence remains unclear on (1) how large the I-GID burden is among preschool children in PICTS, and (2) which specific AE risk factors are associated with I-GID among preschool children in PICTS. This uncertainty may constrain surveillance evaluation and hinder the development of targeted I-GID prevention and control strategies for preschool children in PICTS.

Therefore, this review aimed to provide an overall picture of existing evidence on the burden of I-GID and AE risk factors among preschool children in PICTS. Specifically, we searched for:

1. The extent of the I-GID burden among preschool children in PICTS.
2. Relationships between AE risk factors and I-GID.
3. Key contextual factors (e.g. cultural beliefs and temperature) that may influence the relationship between AE risk factors and I-GID.
4. Knowledge gaps and limitations in the existing evidence.

## Methodology

We conducted a narrative review of scientific and grey literature, informed by a systematic search of the available evidence.

### Scope of the review

To ensure conceptual clarity and consistency, the definitions of PICTS, preschool children, I-GID, AE risk factors, and contextual factors used in this review are provided below.

#### PICTS

PICTS are typically characterised by geographic isolation, dispersed populations across multiple islands, and constrained land resources with a strong reliance on marine environments. Despite substantial variation in population size, economic development, and cultural contexts, these shared features can shape exposure patterns relevant to human health [12].

We identified PICTS based on the Pacific Community (SPC) membership list [24], excluding its developed member countries (the United Kingdom, the United States, France, Australia, and New Zealand). We additionally included Hawaiʻi because it is located in the Pacific and shares key characteristics with other PICTS. The 23 included PICTS are organised into three ethno-geographic regions: Melanesia (Fiji, Papua New Guinea, Vanuatu, Solomon Islands, New Caledonia), Micronesia (Marshall Islands, Palau, Guam, Northern Mariana Islands, Nauru, Kiribati, Federated States of Micronesia), and Polynesia (Tuvalu, Cook Islands, Niue, Tokelau, Samoa, Tonga, American Samoa, French Polynesia, Pitcairn Islands, Wallis and Futuna, Hawaiʻi) (Figure 1).

**Figure 1.**
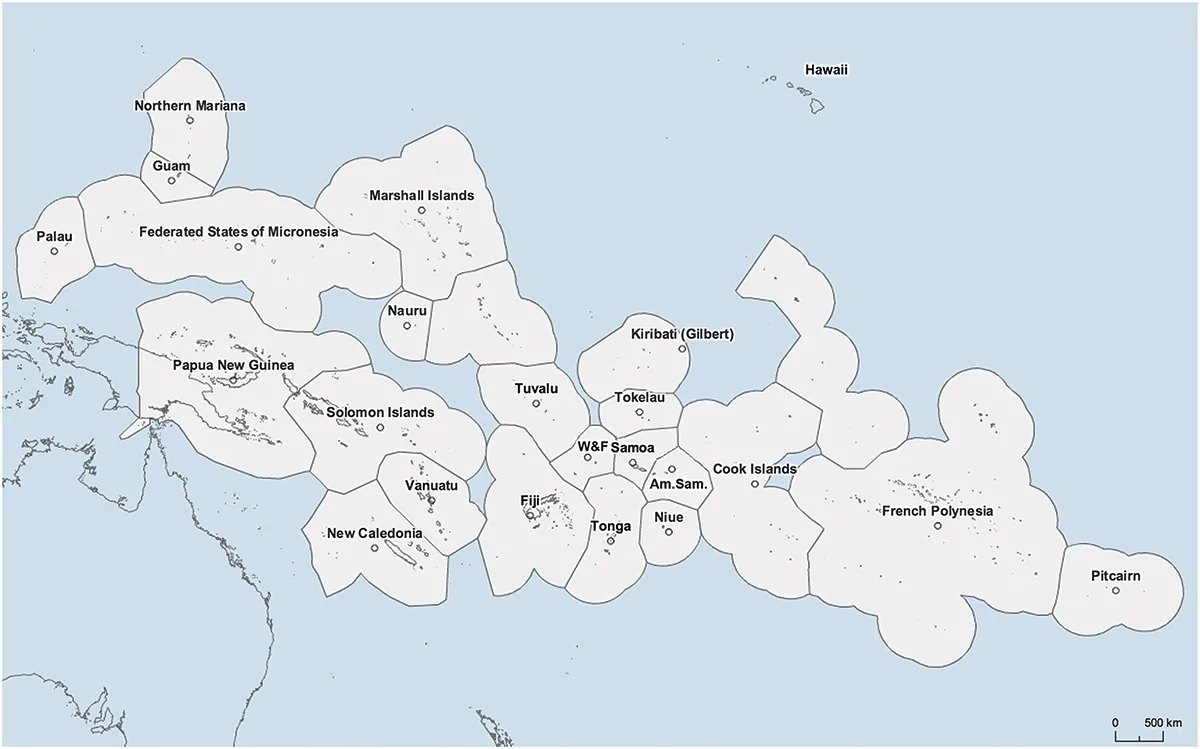
Map of PICTS. Source: Pacific Community (SPC). Reproduced from Guernier et al. [25].

#### Preschool children

The focus population is preschool children, usually defined as those aged 5 years and under (≤5 years). This demographic was selected because preschool children are particularly susceptible to AE risk factors and I-GID [26,27].

#### I-GID

Given the different definitions of I-GID across the literature, this review included and synthesised evidence based on the original case definitions or terms used by the included reports, rather than applying a predefined disease classification.

#### AE risk factors

We defined AE for preschool children as the places and conditions where children spend most of their time living and playing, including domestic environments (housing and adjacent spaces) and institutional environments (preschools and childcare settings).

Two broad types of AE risk factors were considered: (1) *AE-related physical conditions*, which define the environmental context of preschool children’s exposure to I-GID pathogens, such as building conditions, crowding, water supply, sanitation, surrounding environmental hygiene, and animal presence; and (2) *AE-related behaviours and practices*, which directly shape, modify, or maintain these physical conditions or children’s contact with contaminated environments, thereby influencing I-GID pathogen exposure among preschool children. These factors include waste disposal practices, food and water handling practices, and hand-washing behaviours.

Factors that influence I-GID risk through biological or nutritional pathways rather than environmental exposure pathways, such as breastfeeding practices and micronutrient intake (e.g. zinc), were not considered AE risk factors and were therefore excluded from this review.

#### Contextual factors

Contextual factors are external circumstances that do not constitute components of AE itself but may influence the I-GID risk of preschool children by shaping or modifying AE risk factors. These include demographic and socioeconomic factors (e.g. parental education, household income, knowledge, attitude, beliefs, healthcare access, and resources) as well as climatic and broader environmental risk factors (e.g. temperature, rainfall, flooding, and drought).

### Search and review strategy

Given the limited evidence in this setting, a systematic search was conducted to identify relevant literature as comprehensively as possible, followed by a narrative synthesis.

#### Databases

We searched four global academic databases from database inception to 12 May 2025: PubMed, Web of Science, Embase, and Scopus, supplemented by grey literature, including reports from United Nations (UN) agencies (e.g. WHO, UNICEF, United Nations Environment Programme [UNEP]) and PICTS government websites. Reference lists of included reports were reviewed to identify additional literature.

#### Search terms

We used combinations of keywords and their variants relating to the population, exposure, outcome, and setting. Core terms included ‘anthropogenic environment’, ‘risk factors’, ‘burden of disease’, ‘infectious gastrointestinal diseases’, ‘preschool children’, and ‘PICTS’. Where applicable, specific syntax and controlled vocabularies (e.g. MeSH/Emtree) were adapted to each database. No restrictions were applied to language or year of publication. Detailed database-specific search strategies and grey-literature search procedures are provided in Supplementary File 1.

#### Relevance criteria

We reviewed scientific and grey literature relevant to the burden of I-GID and AE risk factors among preschool children in PICTS, including reports providing burden estimates or risk-factor associations, as well as those describing relevant data sources, surveillance systems, and methodological approaches.

To reflect how directly each report addressed the objectives of this review, we developed relevance criteria to grade the retrieved reports as highly relevant, relevant, low relevance, or not relevant, based on the directness of the evidence for the three objectives (Table 1). For Objective 1, only evidence that explicitly reported the disease burden among preschool children was considered direct evidence. For Objectives 2 and 3, evidence from broader populations or based on inference rather than explicitly assessed relationships was considered indirect evidence. Across all three objectives, evidence describing relevant data sources, surveillance systems, or methodological approaches was also considered indirect evidence. Reports classified as not relevant were excluded from the narrative synthesis.

**Table 1.** Evidence definitions and relevance criteria.

Study objectives		Direct evidence		Indirect evidence	
Objective 1: The extent of the I-GID burden among preschool children in PICTS.		Epidemiological characteristics (patterns by gender, age, time, space, pathogens, etc.) and/or economic, social, and health burden of I-GID in preschool children in PICTS.		Evidence on relevant data sources, surveillance systems, and methodological approaches.	
Objective 2: Relationships between AE risk factors and I-GID.		Explicit assessment of the relationship between AE risk factors and I-GID among preschool children in PICTS.		Evidence from broader populations; or the relationships between AE risk factors and I-GID/ the influence of contextual factors were inferred based on risk assessments, risk characterisation, indirect health impact assessments, monitoring and assessment protocols, or conceptual/narrative reports without primary or secondary empirical data; or on relevant data sources, surveillance systems, and methodological approaches.	
Objective 3: Key contextual factors (e.g. cultural beliefs, temperature) that may influence the relationships between AE risk factors and I-GID.		Explicit assessment of whether contextual factors influence the relationships between AE risk factors and I-GID among preschool children in PICTS.			
**Evidence profiles for relevance classification**					
**Level**	**Objective 1**		**Objective 2**		**Objective 3**
Highly relevant	Direct evidence		Direct evidence		Direct, indirect, or none
Relevant	Direct evidence		Indirect or none		Direct, indirect, or none
	Indirect or none		Direct evidence		Direct, indirect, or none
Low relevance^1^	Indirect or none		Indirect or none		Indirect or none
Not relevant^2^	None		None		None

Notes:

1. For low relevance, indirect evidence for at least one of the objectives 1–3 was required.

2. Examples of non‑relevant reports included studies focused on (1) chronic diseases or non-GI infectious diseases; (2) contextual factors without AE; (3) clinical or genomic pathogens without AE drivers; (4) vaccination, treatment, or capacity-building interventions.

#### Screening and data extraction

Following deduplication, reports were screened and graded in two stages using predefined relevance criteria: title and abstract screening, followed by full-text review.

A structured data sheet was used to extract and organise information from included reports, including title, authors, publication years, study regions, study population, data sources, study design, study diseases (definitions, pathogens, diagnosis methods), risk factors, and significant findings. Two researchers (JC and FY) independently conducted screening, grading, and data extraction. All conflicts were addressed by the senior author (PJ).

#### Study synthesis

Findings were categorised into four fundamental themes crucial to understand preschool children’s I-GID and its AE risk factors in PICTS: (1) I-GID definitions, (2) burden of I-GID, (3) AE and contextual risk factors for I-GID, and (4) data sources and methodological approaches. These themes provided the structure for synthesising evidence and identifying key knowledge gaps.

## Findings and discussion

### Overview of included reports

The systematic search retrieved 8,582 reports; 4,337 duplicates were removed, leaving 4,245. After full-text screening and grading, 48 reports were retained, all in English. The search flow diagram is shown in Figure S1, Supplementary File 2.

The main characteristics of the included reports are summarised in Table 2, with detailed information on individual reports provided in Table S1, Supplementary File 3. The reports came from 12 PICTS, with 15 (31.3%), 7 (14.6%), 6 (12.5%), and 4 (8.3%) from Papua New Guinea, the Solomon Islands, Fiji, and Vanuatu, respectively, and three (6.3%) covered multiple PICTS settings. Eleven PICTS (New Caledonia, Palau, Guam, Northern Mariana Islands, Federated States of Micronesia, Cook Islands, Niue, Tokelau, American Samoa, French Polynesia, Pitcairn Islands) had no reports. The included reports were published between 1972 and 2024, with 20 (41.7%) published after 2014. Forty‑five (93.8%) reports were scientific journal articles, and the other three (6.3 %) were from grey literature. Thirty‑three (68.8%) reports examined the burden of I-GID among preschool children in PICTS, 26 (54.2%) investigated associated risk factors, including 22 (45.8%) examining AE risk factors and 8 (16.7%) examining contextual factors. Five (10.4%) reports provided information on relevant data sources. Of all included reports, only six (12.5%) were classified as highly relevant, providing direct evidence on both I-GID burden and AE risk factors. Twenty‑nine (60.4%) reports were classified as relevant, and 13 (27.1%) as low relevance. The list of all included reports is shown in Table S2, Supplementary File 3.

**Table 2.** Characteristics of included reports (*N* = 48).

Categories	Number of reports (%)
Study location	
Papua New Guinea	15 (31.3)
Solomon Islands	7 (14.6)
Fiji	6 (12.5)
Vanuatu	4 (8.3)
Samoa	2 (4.2)
Kiribati	2 (4.2)
Marshall Islands	2 (4.2)
Hawaiʻi	2 (4.2)
Tuvalu	2 (4.2)
Tonga	1 (2.1)
Wallis and Futuna	1 (2.1)
Nauru	1 (2.1)
PICTS	3 (6.3)
Publication years	
2015-current	20 (41.7)
Before 2015	28 (58.3)
Report types	
Scientific journal article	45 (93.8)
Grey literature	3 (6.3)
Category of results*	
Burden of disease	33 (68.8)
Any risk factors	26 (54.2)
AE risk factors	22 (45.8)
Contextual factors	8 (16.7)
Data availability	5 (10.4)
Relevance	
Highly relevant	6 (12.5)
Relevant	29 (60.4)
Low relevance	13 (27.1)

*The sum of ‘Category of results’ exceeds the total number of included reports, as some reports covered multiple outcome types.

### Diseases reported with definitions

#### Findings

Table S3 (Supplementary File 3) summarises the I-GID case definitions used in the included reports. Forty‑four reported a specific I-GID outcome, while four did not directly assess a specific I-GID. In most reports (*n* = 30, 68.18%), I-GID was defined by symptom-based criteria, either alone or followed by pathogen identification. Among these, diarrhoea was the most used term (*n* = 26), largely based on modified WHO definitions. Gastroenteritis was another definition for I-GID (*n* = 4). Four of the symptom-based reports further used targeted diagnostics to identify specific pathogens, most commonly rotavirus, via stool testing (see Burden of I-GID in preschool children in PICTS). Furthermore, 14 (31.82%) reports focused on specific infectious diseases, including typhoid fever (*n* = 6), salmonellosis (*n* = 2), soil-transmitted helminth (STH) infections (*n* = 5), and cholera (*n* = 1), which were predominantly diagnosed using laboratory-confirmed pathogen-specific methods (Table S3, Supplementary File 3).

#### Discussion

Overall, most reports of I-GID among preschool children in PICTS used syndromic case definitions, primarily diarrhoea, rather than focusing on specific I-GID. Given limited health resources and diagnostic laboratory capacity in PICTS [28], syndrome definitions help identify suspected cases and guide appropriate treatment. However, diarrhoea is a non-specific symptom that can arise from multiple infectious and non-infectious causes, and some I-GID may present without diarrhoea [29–31]. Consequently, reliance on syndromic definitions may reduce the accuracy of burden estimates and limit the ability to identify specific pathogens, risk factors, and transmission pathways, thereby constraining the development of targeted prevention and control strategies. Additionally, case definitions varied across the included reports for both syndrome and specific I-GID. This heterogeneity reduces comparability across reports and limits the transferability of evidence from one context to another.

### Burden of I-GID in preschool children in PICTS

#### Findings

Thirty three reports (68.8%) described the burden of I-GID (Table S4, Supplementary File 3), with 12 focusing on diarrhoea as symptomatic of I-GID. Incidence estimates of diarrhoea were reported in three PICTS (Nauru, Papua New Guinea, and Fiji), using heterogeneous measures. Reported rates included 510.7 per 1,000 children during outbreaks, 0.17–3.4 episodes per 100 child-weeks, 3.0 episodes per child-year, and 1,251 per 100,000 population per year (ID45, 8, 41, 25). Within the preschool‑age group, the highest incidence was generally observed among children under 3 years, although age groupings varied across reports. The prevalence of diarrhoea in Fiji was 12.3% (ID43). Four reports (ID32, 13, 2, 14) assessed the prevalence of self-reported diarrhoeal cases, although recall periods varied across reports. Furthermore, based on Multiple Indicator Cluster Survey (MICS) data (ID47), self-reported diarrhoeal prevalence ranged from 3.3% to 18.2% in seven PICTS between 2018 and 2023. Attack rates (incidence during an outbreak) ranged from 12% to 32% across Tuvalu, Solomon Islands, and the Marshall Islands (ID12, 26, 37). Several reports also reported a case fatality rate of 3.9% in Tonga (ID10), a proportional mortality of 5.6% in Vanuatu (ID9), and a hospitalisation proportion of 2.0% in Tuvalu (ID12). One report (ID38) also showed an attack rate of 13.0% for gastroenteritis in Kiribati.

Typhoid fever, STH infection, and salmonellosis metrics were also reported. Incidence of typhoid fever in Fiji and Samoa was 19 per 100,000 (ID15, 39) and 25.0–40.9 per 100,000 (ID36). These reports also indicated that preschool children had the lowest incidence among age groups. However, STH infection led to a high burden of I-GID among preschool children in PICTS, with the prevalence of any STH infection ranging from 41% to 100% (ID5, 17, 27, 28, 44). Two reports (ID16, 42) from Hawaiʻi reported salmonellosis incidence ranging from 95 to 323 per 100,000/year; however, these data were collected prior to 1990.

Several reports described the burden of specific pathogens contributing to I-GID. A report (ID25) in Fiji in 2009 found that rotavirus was the main cause of diarrhoea in preschool children, with an admissions rate of 486 per 100,000/year. Three additional reports (ID21, 26, 35) from the Solomon Islands and Papua New Guinea showed that rotavirus accounted for more than 30% of all diarrhoea cases among preschool children. *Shigella* infections accounted for more than 20% of all diarrhoea cases among preschool children (ID18, 30). Additional epidemiological and clinical findings are provided in Table S4, Supplementary File 3.

#### Discussion

According to Global Burden of Disease (GBD) 2023 estimates, the diarrhoea DALY rate among preschool children ranged from 142.99 per 100,000 in Tonga to 3,741.12 per 100,000 in Kiribati, which were all below the global estimate (4,501.29 per 100,000) [32]. However, our review identified limited primary data on I-GID burden among preschool children in PICTS. In data-sparse settings, GBD estimates rely heavily on statistical modelling and extrapolation, and therefore may not fully capture the true burden in specific PICTS contexts [7]. Nevertheless, rotavirus was found as a leading cause of I-GID among preschool children in PICTS in the included reports, consistent with global evidence [33].

Studies from other LMICs have shown that I-GID among preschool children is closely associated with poverty and can impose substantial socioeconomic burdens, including household out-of-pocket expenditure, caregiver productivity losses, and increased health-system utilisation. Recurrent or persistent diarrhoeal illness may also have longer-term consequences for child development and human capital formation [34–42]. However, none of the included reports assessed the socioeconomic burden of I-GID among preschool children in PICTS. In this resource-limited region, the lack of local economic evidence hampers robust cost-effectiveness analyses and health technology assessments, thereby constraining evidence-based resource allocation and the prioritisation of prevention and control strategies.

Overall, the evidence on I-GID burden among preschool children in PICTS remains limited and is often outdated. Considerable heterogeneity in case definitions and outcome measures further reduces comparability across reports. These gaps make it difficult to reliably assess the burden of I-GID among preschool children in PICTS based on existing evidence.

### Risk factors for I-GID in preschool children in PICTS

Twenty six reports provided evidence on AE (22) or contextual factors (8) relevant to I-GID in PICTS. Detailed information is shown in Table S5, Supplementary File 3.

#### Findings

##### AE risk factors

Evidence on AE-related physical conditions was concentrated in water, sanitation, waste and surrounding environment, housing, and animals. Only three reports (ID8, 13, 14) provided direct evidence on associations between household water factors and diarrhoea among preschool children, with inconsistent findings: two reports (ID8, 13) found associations with water accessibility or household water source, whereas the third (ID14) found no association with household water source. In addition, seven reports (ID12, 20, 23, 28, 31, 32, 33) provided indirect evidence on household water factors and diarrhoea, typhoid fever, or STH infection. Water insufficiency and drinking-water contamination were associated with I-GID, whereas findings for household water source were inconsistent. ID32 demonstrated differences in boiled drinking water and source water contamination but did not directly link contamination to diarrhoea.

Three reports (ID8, 14, 27) provided direct evidence but found no association between sanitation facility type and preschool children’s diarrhoea or STH infection. However, three indirect reports (ID23, 28, 31) found that unimproved sanitation facilities, poorer sanitary conditions, or limited sanitation availability were associated with an increased risk of typhoid fever or STH infection, while ID33 provided only descriptive evidence on sanitation conditions.

Two reports (ID8, 14) provided direct evidence and found that adverse surrounding AE conditions were associated with an increased risk of diarrhoea in preschool children. Three other reports provided indirect evidence on surrounding AE: seawater-related transmission pathways were proposed, but no direct links were examined (ID5, 30), while toilet-drainage soil contamination was associated with typhoid fever (ID23) and less acidic soil with higher STH infection risk (ID28).

One report (ID8) found that the presence of pigs near the household was associated with an increased risk of diarrhoea in preschool children, whereas another mixed-age report (ID12) found no association between livestock ownership and diarrhoea.

One report (ID27) found that household size and traditional housing construction types were associated with an increased STH infection risk in preschool children, although the associations varied across STH species. Several reports examined composite indicators related to AE but provided only indirect evidence. ID41 defined living conditions using a combination of household size, electricity and water availability, and latrine type and found that preschool children with poorer living conditions had a higher incidence of diarrhoea. ID23 grouped multiple individual AE variables into five categories: external conditions, drinking water conditions, sanitary conditions, soil microbial loads, and soil nutrient loads. These composite indicators were associated with typhoid fever.

For AE-related behaviours and practices, evidence concerned water, sanitation, waste and the surrounding environment, animals, food, and hygiene. Four reports (ID2, 7, 24, 32) provided direct evidence on associations between AE-related behaviours and practices and diarrhoea in preschool children. Findings from these reports showed that river water use for bathing and laundry, open defecation by children, improper disposal of children’s faeces, sleeping with pigs, and consumption of leftover food or food from outside the household were associated with a higher risk of diarrhoea. In contrast, other practices, including inappropriate disposal of solid waste, boiling water before drinking, and regular toilet cleaning, were not found to be associated with diarrhoea. However, evidence on caregiver handwashing behaviour was inconsistent across reports (ID2, 32).

Indirect evidence on AE-related behaviours and practices and I-GID, including diarrhoea, cholera, typhoid fever, and STH infection, was provided in nine reports (ID12, 19, 22, 28, 30, 31, 34, 43, 46). Open defecation was associated with cholera in ID46, whereas eating unwashed produce was associated with typhoid fever in ID31. Handwashing or soap availability was associated with a lower risk of diarrhoea, typhoid fever, or cholera in ID12, 31, and 46, while wearing shoes outdoors was associated with a lower risk of STH infection in ID28. A combined filtration and handwashing intervention was also followed by substantially lower diarrhoea prevalence (ID43). An outbreak investigation implicated food handling and storage practices but did not identify any individual food item as statistically associated with gastroenteritis (ID19). Environmental detection of pathogens in seawater provided only indirect evidence of potential food-related transmission (ID30). Two additional reports (ID22, 34) focused on child faeces management rather than directly assessing I-GID outcomes. Unsafe disposal was commonly reported, and child faeces disposal practices were associated with behavioural, infrastructural, demographic, environmental, and socioeconomic factors.

##### Contextual factors

Eight reports examined contextual factors, but all provided indirect evidence. None formally assessed whether these contextual factors modified or mediated the relationship between AE risk factors and I-GID.

Younger maternal age, lower birth order, and lower maternal education were associated with a higher risk of diarrhoea and STH infection among preschool children (ID24, 27). A qualitative report showed that some locals attributed I-GID to cultural beliefs and developmental stages rather than pathogen ingestion, which may be related to fewer safe hygiene practices (ID48). A narrative review in PICTS showed that unplanned urbanisation contributed to slum formation, poor sanitation, and overcrowding, which may increase preschool children’s susceptibility to I-GID (ID6). Another report found that children’s age and caregivers’ age and gender were associated with faeces-handling practices, and that poor disposal contaminated the environment, potentially increasing I-GID risk among preschool children (ID34).

PICTS are highly vulnerable to rapid climate change, which increases the risk of I-GID in preschool children by altering AE. In Tuvalu, a diarrhoea outbreak occurred during a severe drought that reduced household water availability and hygiene practices (ID12). Two reports described disruption to water, sanitation, housing, and living conditions following tsunamis, with potential implications for I-GID transmission (ID4, 29). A narrative review (ID6) highlighted that changes in average annual temperature and extreme rainfall (either excessive or insufficient) disrupted water security through altered precipitation patterns, droughts, and sea‑level‑rise-induced salinisation. It also suggested that floods and storms may damage housing and increase overcrowding, thereby facilitating I-GID transmission among preschool children.

#### Conceptual diagram and discussion

Based on the included reports, a conceptual diagram was developed to summarise risk factors potentially associated with I-GID among preschool children in PICTS (Figure 2). The diagram illustrates possible factors without implying causality or the direction of effects, and all risk factors shown are derived from the included evidence.

**Figure 2.**
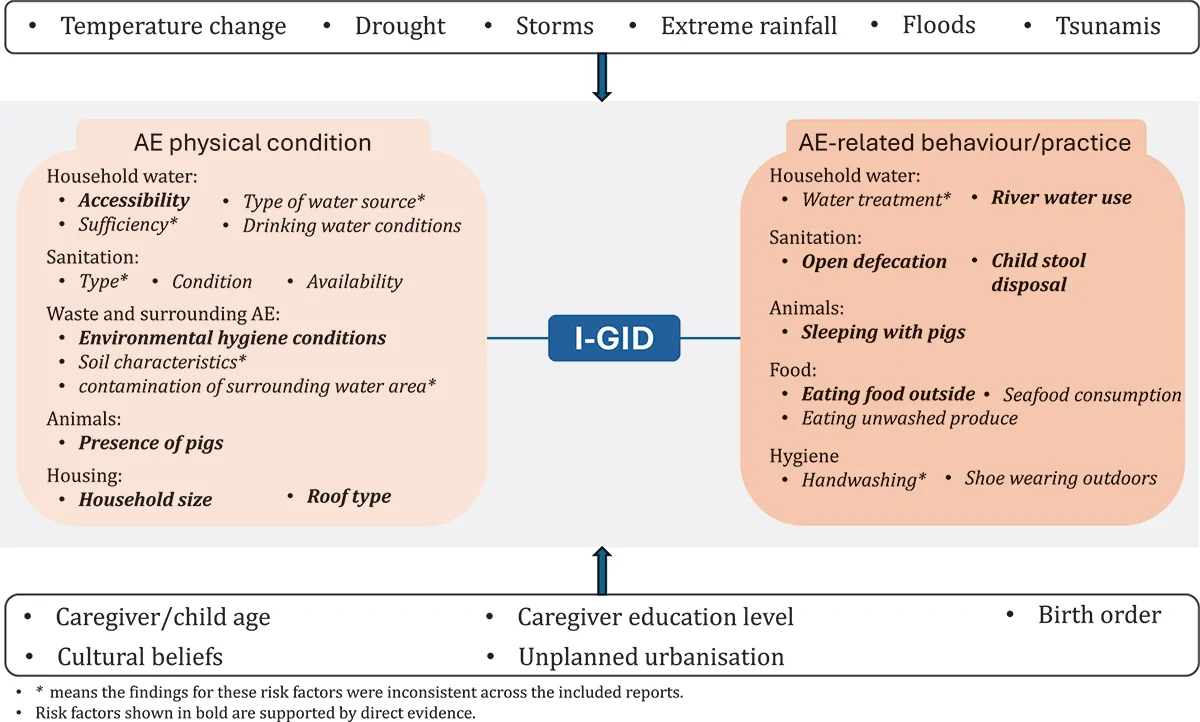
Conceptual diagram of the relationship between I-GID and its risk factors among preschool children in PICTS.

Across the included reports, several AE risk factors were identified from direct preschool-specific evidence. These spanned both physical environmental conditions, including water accessibility, the surrounding environment, animal presence, and housing, and behaviours that increase faecal–oral exposure, including child open defecation, child faeces disposal, river water use, contact with animals, and food consumption. Taken together, these findings suggest that I-GID risk among preschool children in PICTS is associated with both physical environmental conditions and behaviours that increase opportunities for faecal–oral exposure. However, some AE risk factors commonly associated with I-GID in LMICs, such as sanitation facility type, water treatment, and handwashing behaviours [43–45], showed inconsistent findings in the included reports.

I-GID transmission primarily occurs through three environmental faecal–oral transmission pathways: contaminated water, contaminated food, and contaminated hand-to-mouth contact, each influenced by distinct AE risk factors [46]. However, no report has examined the movement of contaminants arising from AE risk factors along these pathways. We noted that a few reports (ID5, 23, 28, 30, 43) measured contamination in soil, water sources, and surrounding waters, but none linked these measurements to I-GID outcomes; hence, the main transmission pathways cannot be clearly identified. Overall, due to limited evidence and insufficiently rigorous and comprehensive methods, the AE risk factors of I-GID among preschool children in PICTS remain poorly understood.

Evidence on contextual factors shaping the AE–I-GID relationship was also scarce. Globally, socioeconomic and demographic factors were found to shape AE risks and affect the effectiveness of AE-related interventions or exposure behaviours related to contaminated AE [47–50], consistent with findings from our included reports, including parental education and urbanisation. Socioeconomic inequalities shape both AE risks and the capacity to avoid and manage them, contributing to unequal I-GID risks [51]. However, the available evidence was too sparse and heterogeneous to quantify socioeconomic gradients in I-GID risk across populations and regions in PICTS.

Regarding climatic and broader environmental risk factors, PICTS are among the most vulnerable regions to rapid global climate change [20,52,53]. Although many reports in PICTS linked climate change to I-GID [14,54], few examined how climate change may affect AE risk factors and thereby shape I-GID risk, particularly among preschool children. Therefore, while an association between climate change and I-GID is suggested, the pathways linking climate change, AE risk factors, and I-GID remain unclear.

Integrating evidence on risk factors with health and economic burden estimates is important for investment prioritisation. Studies from other LMICs have shown that sanitation and drinking-water supply interventions can be cost-effective and provide favourable economic returns [55]. However, as one of the regions most vulnerable to environmental and socioeconomic shocks, PICTS may face different risks from other regions [56]. Although investments have been made in I-GID prevention and control, such as implementing WASH programmes [22], the limited evidence on I-GID risk factors in PICTS makes it difficult to determine which intervention yields the greatest health gain per dollar invested. Therefore, a systematic assessment that combines the burden of diarrhoeal diseases, the identification of relevant risk factors, and the cost-effectiveness analysis of addressing these risk factors and existing investments is particularly important for PICTS.

### Data sources and methodological approaches applied in preschool children with I-GID studies in PICTS

The reviewed reports indicate that data resources for I-GID among preschool children in PICTS are limited, suggesting relatively weak disease surveillance and environmental monitoring capacity. One report (ID3) from Papua New Guinea noted that logistical and funding constraints are key challenges for strengthening surveillance systems. Within the included reports, national infectious disease surveillance systems that included diarrhoea syndromic surveillance were reported only for Papua New Guinea, Solomon Islands, and Vanuatu, and these systems were established around 2013 (ID3, 4, 40). As of 2013, systematic typhoid surveillance covering all age groups was reported in only Fiji, Samoa, and Palau (ID11).

The PSSS, in operation since 2010, conducts weekly surveillance of four syndromes, including diarrhoea, using data from sentinel health facilities across 23 PICTS. However, PSSS data do not include age-specific information, limiting their utility for assessing preschool I-GID burden. Apart from PSSS, we could not identify any accessible reports describing I-GID surveillance systems in other PICTS. In addition to the above surveillance systems, the MICS (ID47) provides nationally representative household survey data; however, data are available for only seven PICTS, and the survey focuses on self-reported diarrhoea prevalence rather than specific I-GID. Notably, MICS was the only publicly accessible data source identified in this review to include information relevant to AE monitoring in PICTS.

In the reviewed scientific journal articles, most reports on I-GID used descriptive analyses to assess burden, with surveillance data from health facilities. Some reports conducted community-based surveys to estimate self-reported prevalence. No reports applied more advanced modelling techniques to quantify the I-GID burden with relatively lower uncertainty or to investigate its spatiotemporal trends.

For risk factors, most reports collected data through household assessments. Traditional statistical approaches, such as logistic and Poisson regression models, were used to analyse I-GID risk factors. A few reports (ID5, 28, 30) on integrated environmental microbiology tested environmental and faecal samples to infer transmission pathways, but did not focus only on preschool children. Additionally, some reports focused on environmental assessments, documenting the prevalence of risk factors at the community level without directly linking them to I-GID. Notably, a 1993 report from Papua New Guinea used ethnographic methods and structured observation to explore a wide range of variables (ID24). While this approach captures sociocultural context and provides more accurate behavioural data, its findings are difficult to compare with conventional epidemiological studies.

## General discussion

This review synthesised existing evidence on the burden of I-GID among preschool children in Pacific Island Countries, Territories, and States (PICTS), and the anthropogenic environment (AE)risk factors and broader contextual factors that may influence I-GID risk. We identified 48 reports published across 12 PICTS between 1972 and 2024; one-third (31.3%) were from Papua New Guinea, and only six (12.5%) were highly relevant to the review aim.

Regarding the disease burden of I-GID, the included reports used heterogeneous I-GID definitions and a wide range of health burden metrics, limiting direct comparison. Overall, the I-GID burden in PICTS was reported to be lower than the global average but varied across locations and I-GID conditions[7]. However, the reported burden may not reflect the true burden in this region. First, multiple I-GID outbreaks were reported across PICTS, with preschool children disproportionately affected (ID26, 37, 38, 45). Second, five reports (ID5, 17, 27, 28, 44) suggested a substantial I-GID burden associated with soil-transmitted helminth infections among preschool children in PICTS, with prevalence ranging from 41% to 100%. Third, for specific pathogens, two reports (ID15, 25) highlighted barriers to paediatric specimen collection and limited laboratory capacity in PICTS, suggesting that pathogen-specific burden in preschool children may be underdetected. Taken together, and considering well-recognised constraints on surveillance and diagnostic systems in many PICTS [28], it is likely that I-GID among preschool children in PICTS is underdetected and underreported and that the actual burden is still uncertain. Such uncertainty and surveillance limitations have implications beyond epidemiological assessment, creating both epidemiological and economic inefficiencies by delaying outbreak detection, misdirecting limited health resources, and weakening the evidence base for infrastructure investment and prevention planning.

Regarding I-GID risk factors, the available evidence suggests that multiple AE domains are relevant to I-GID among preschool children in PICTS, but the evidence remains sparse and heterogeneous. Many potential AE risk factors and transmission pathways have not been adequately assessed, and some findings rely on indirect evidence from mixed-age populations or descriptive and non-associational analyses. Several included reports discussed contextual factors, including climate change and cultural and socioeconomic conditions, that could influence I-GID risk in PICTS. However, this evidence was entirely indirect, and none of the included reports formally assessed whether broader contextual factors modify, mediate, or amplify AE–I-GID relationships. Evidence on risk factors was also geographically concentrated, particularly in Papua New Guinea and the Solomon Islands. Given the substantial heterogeneity across PICTS in cultural, economic, and natural contexts [12], AE risk factors and transmission dynamics are likely to differ across PICTS, but this could not be reviewed from the current evidence.

This review has several limitations. First, the included reports showed substantial heterogeneity in disease definitions, methodological rigour, and findings, and more than half were published before 2015. We also did not conduct a formal quality or risk-of-bias appraisal. These inconsistencies limited our ability to compare and synthesise findings across reports, generate pooled estimates for specific outcomes, and identify clear causal pathways or transmission routes. Our synthesis of disease burden and of risk-factor associations is therefore descriptive. Second, the available evidence was geographically uneven, with more than half coming from Papua New Guinea, the Solomon Islands, and Fiji, while 11 PICTS had no identified reports. Given the substantial socioeconomic, cultural, geographic, and environmental variation across PICTS, our findings may not be representative, and their applicability to other PICTS settings is limited. Third, some included reports of risk factors involved broader age groups rather than preschool children specifically, which limits the direct applicability of these findings to preschool children. Fourth, despite extending the search beyond scientific and English-language literature, some local reports in other languages may have been difficult to access, particularly across small island settings, and relevant local evidence may have been missed. Finally, publication bias and selective outcome reporting resulted in studies with statistically significant or positive findings being more likely to be published or accessible than those reporting null or negative findings, reflecting limitations of the current evidence base rather than the absence or unimportance of the risk factors concerned.

## Conclusion

Although the burden of I-GID among preschool children in PICTS appears to be lower than the global average, it is still inadequately characterised. There is also an apparent evidence gap between AE contamination or exposure and demonstrated infection transmission, as well as limited evidence on the role of contextual factors in shaping AE–I-GID relationships. Besides, I-GID burden and AE risk factors appear to vary widely across I-GID types and geographic regions. However, due to limited local data, inconsistent case definitions, and insufficiently rigorous or comprehensive methods, the magnitude of these differences remains unclear. Given the socio-environmental challenges faced by PICTS, our understanding of the I-GID burden and its risk factors in this region requires urgent upscaling. More research integrating environmental, microbiological, epidemiological, and surveillance approaches is needed to better characterise disease burden, transmission pathways, and key AE and contextual risk factors, thereby supporting timely and effective interventions and control strategies in PICTS.

## Supplementary Material

Supplementary File 3 Characteristics and evidence tables of included reports.xlsx

Manuscript_clean.docx

Supplementary File 2 Study screening and selection flow diagram.docx

Supplementary File 1 Search strategies.xlsx
