# Supplementary material for: Anthropogenic environment and the burden of infectious gastrointestinal diseases in preschool children in Pacific Island countries, territories, and States: a narrative review
Source: Glob Health Action. 2026 Oct 22;19(1):2750012. doi: 10.1080/16549716.2026.2750012 (PMC13700472; doi:10.1080/16549716.2026.2750012)
Supplement: Supplementary file 1 [file ZGHA_A_2750012_SM8204.docx]

Reports from other sources **(n =346)**

**Identification**

Title and abstract screen **(n = 4,245)**

Reports retrieved **(n = 388)**

Reports assessed for eligibility **(n = 345)**

Duplicates removed **(n = 4,337)**

Reports excluded **(n = 3,857)**

Reports not retrieved **(n = 43)**

**Screening**

Reports from databases/registers **(n = 8,236)**

PubMed (n = 1,671)

Web of Science (n = 505)

Embase (n = 4,323)

Scopus (n = 1,737)

Reports excluded **(n = 297)**

Not PIC (n = 120)

Not GID (n = 62)

Population not relevant (n = 44)

Topic outside review scope (n = 32)

Duplicates (n=39)

Reports included **(n = 48)**

**Included**

**Figure S1. Search flow diagram**
